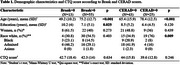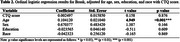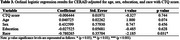# *Childhood trauma and anatomopathological changes*.

**DOI:** 10.1002/alz70855_102228

**Published:** 2025-12-23

**Authors:** Salma Rose Imanari Ribeiz, Leonardo Baracat Caria, Wanessa Santos, Carlos Augusto Pasqualucci, Ricardo Nitrini, Eduardo Ferrioli, Lea T. Grinberg, Renata Elaine Paraizo Leite, Paula Villela Nunes, Camila Nascimento Mantelli, Claudia Kimie Suemoto

**Affiliations:** ^1^ Faculdade de Medicina de Jundiaí, Jundiaí, Sao Paulo, Brazil; ^2^ Institute of Psychiatry, Faculty of Medicine, University of São Paulo, Sao Paulo, Sao Paulo, Brazil; ^3^ Universidade Federal de São Paulo, Sao Paulo, Sao Paulo, Brazil; ^4^ Department of Pathology, University of São Paulo Medical School, São Paulo, São Paulo, Brazil; ^5^ Department of Neurology, Faculdade de Medicina FMUSP, Universidade de Sao Paulo, Sao Paulo, Sao Paulo, Brazil; ^6^ Division of Geriatrics, Department of Internal Medicine, University of São Paulo Medical School, São Paulo, São Paulo, Brazil; ^7^ Memory and Aging Center, UCSF Weill Institute for Neurosciences, University of California, San Francisco, San Francisco, CA, USA; ^8^ University of British Columbia, Vancouver, BC, Canada

## Abstract

***Relationship between childhood trauma and Alzheimer´s disease pathological changes in a psychiatric sample***.

**Background:**

The stress resulting from traumatic events throughout life is often associated with an increased risk of developing mental disorders. The damaging effects of childhood trauma are widely presented in specific psychiatric conditions. Some studies suggest the potential occurrence of neurocognitive deficits in individuals exposed to adverse childhood experiences. To our knowledge there are no studies investigating neuropathological changes in individuals exposed to early trauma. This is an original study that aims to assess the possible association between the occurrence of childhood trauma and the development of Alzheimer´s disease anatomopathological (AP) changes.

**Methods:**

We included 68 participants of this study from Biobank for Aging Studies of the University of Sao Paulo (BAS‐USP). Subjects were previously diagnosed with a psychiatric disorder using Structured Clinical Interview for DSM‐IV Disorders (SCID) for Axis I, informant part. Childhood trauma was assessed with the Childhood Trauma Questionnaire (CTQ). AD‐type neuropathology was evaluated using the Consortium to Establish a Registry for AD (CERAD) criteria for neuritic plaque burden, and the Braak and Braak staging for neurofibrillary tangle pathology. The cohort was dichotomized into 2 groups: Braak stage 0 vs. Braak stage >0 and CERAD stage 0 vs.CERAD >0. Demographic and clinical characteristics were compared between groups using the Student's t‐test, Mann‐Whitney U test, chi‐square test, or Fisher's exact test. To investigate the association of childhood trauma (measured by the total score of CTQ) with AP neuropathology, we employed multivariable ordinal logistic regression models for both Braak and CERAD stages, adjusted for age, sex, education, and race.

**Results:**

Demographic analysis (Table 1) showed that younger age was associated with Braak=0 (*p* <0.001) and CERAD=0 (*p* <0.001). Braak=0 was also associated with a higher level of education (*p* = 0.025). The CTQ score did not show a significant effect on Braak and CERAD scores (Tables 2 and 3, respectively).

**Conclusion:**

Our preliminary data does not support a relationship between AD‐related pathology and childhood trauma in a psychiatric sample. Subsequent studies with greater sample size are encouraged to better investigate whether there is a link between AD‐type neuropathology and childhood traumatic experiences.